# SecA—a New Twist in the Tale

**DOI:** 10.1128/JB.00736-16

**Published:** 2016-12-28

**Authors:** Ian Collinson

**Affiliations:** School of Biochemistry, University of Bristol, Bristol, United Kingdom; Princeton University

**Keywords:** protein translocation, SecY complex, bacterial secretion, ATPase, protein targeting

## Abstract

A paper published in this issue of the *Journal of Bacteriology* (D. Huber, M. Jamshad, R. Hanmer, D. Schibich, K. Döring, I. Marcomini, G. Kramer, and B. Bukau, J Bacteriol 199:e0622-16, 2017, https://doi.org/10.1128/JB.00622-16) provides us with a timely reminder that all is not as clear as we had previously thought in the general bacterial secretion system. The paper describes a new mode of secretion through the Sec system—“uncoupled cotranslocation”—for the passage of proteins across the bacterial inner membrane and suggests that we might rethink the nature and mechanism of the targeting and transport steps toward protein export.

## INTRODUCTION

Here we go again—there's yet another talking point on the age-old problem of SecA. SecA is the conserved ATPase found in all bacteria and chloroplasts (but not mitochondria) that is responsible for ATP and proton motive force (PMF)-driven secretion of proteins through the SecYEG complex at the bacterial inner membrane or the plant thylakoid membrane. A collective *tour de force* of genetics and biochemistry within the laboratories of Wickner, Silhavy, Beckwith, and Ito revealed the principle components of the bacterial protein secretion machinery: the protein channel complex SecYEG, the ancillary subcomplex SecDF-YajC, SecB, and the ATPase motor protein SecA itself (1–13). Later on, YidC joined the party in the aid of membrane protein insertion (14).

**FIG 1:**
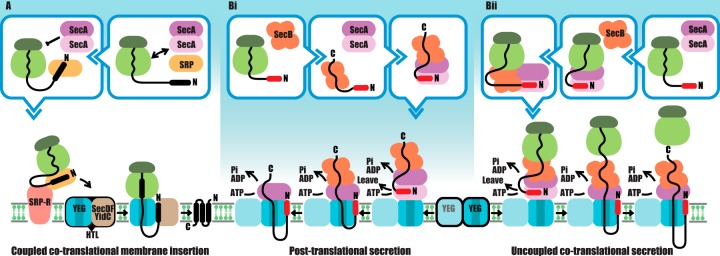
Schematic overview of post- and cotranslational secretion and membrane protein insertion after Huber et al. (39). (A) Coupled cotranslational translocation. Nascent membrane proteins are sampled by SecA and SRP; the latter wins this contest and escorts the ribosome nascent chain (RNC) via its receptor at the cytosolic membrane. The RNC is then transferred to the holotranslocon (HTL)—an assembly of the protein channel complex (SecYEG), the ancillary subcomplex SecDF-YajC (SecDF), and the facilitator of membrane protein insertion, YidC. The processive power of protein synthesis is directly coupled to membrane insertion. In some instances, SecA might cooperate in this venture; SecA is certainly capable of forming a productive association with HTL, as well as SecYEG (B) (19) (Bi) Posttranslational translocation. The classical pathway, whereby presecretory proteins, with cleavable signal sequences (red rod), associate with SecB and SecA, which maintains an unfolded translocation-competent state. Docking of the complex with SecYEG results in SecA dimer dissociation, activation of the ATPase activity, preprotein intercalation, and transport (47–49). (Bii) Alternative uncoupled cotranslational translocation. SecA and SecB associate with nascent secretory proteins and usher the RNC to the SecYEG complex. As described in the legend to panel Bi, SecA dimer dissociation, ATPase activation, and intercalation of unfolded polypeptide bring about translocation. Protein synthesis, disconnected from transport, continues and ends, and posttranslational translocation ensues. The preference for posttranslational (Bi) or uncoupled cotranslational translocation (Bii) will depend on the relative and variable rates of protein synthesis and protein translocation.

The core translocon is formed by SecYEG, supported by a second, nontranslocating (and nonessential [15]) copy (16, 17) for SecA-driven secretion (9). SecYEG also associates with SecDF-YajC and YidC to form the holotranslocon for efficient membrane protein insertion and assembly (1, 18–20). For many years, we have come to accept a simple scheme whereby membrane proteins are targeted to the membrane by the signal recognition particle (SRP) and its receptor (21–27) prior to handover to the translocon for insertion into the membrane via SecYEG and YidC during protein synthesis—cotranslationally (Fig. 1A). In contrast, the translocation of periplasmic and outer membrane proteins is driven by SecA through SecYEG after protein synthesis is complete—posttranslationally (Fig. 1Bi). In both cases, the polypeptide, be it a presecretory protein with a cleavable N-terminal signal sequence or an α-helical transmembrane protein, is threaded across or into the membrane in an unfolded conformation. This translocation-competent conformation is maintained either by the confines of the ribosome and translocon during insertion or by a chaperone such as SecB. In Escherichia coli, it is accepted that the preprotein is shuttled along a cascade involving first SecB, followed by SecA and SecYEG (Fig. 1Bi) (28).

In recent years, and within this framework, the mechanism of protein secretion and insertion has been addressed through the painstaking determination of the structures of all of the key components: SecA (29), SecYEG (30, 31), SecDF (32), YidC (33), and SecB (34), along with the description of the architecture of a number of structures of translating ribosomes associated with the signal recognition elements (35, 36) and with SecYEG (37, 38). There is indeed a great deal of information with which to consider the molecular mechanism of the secretion and insertion process. And still, we have yet to solve this outstanding problem. So it is with great interest we find, in an article in the current issue of the *Journal of Bacteriology* (39), that the overriding view of the pathway leading to secretion and insertion may not be quite right. Therefore, it may be time to reconsider precisely how the various factors combine and operate to bring about the efficient translocation of polypeptides across and into the membrane.

Huber's new paper (39) builds on previous work on the curious interaction between SecA and the ribosome (40, 41), which does not seem to fit the classical view described above. The interaction is at the busy exit site, where it must compete for access to the nascent chain with SRP (36, 42). This raised interesting questions about the role of SecA in cotranslational translocation, which the current work explores. Presumably, the competition at the ribosome exit site is decided by the affinity of the nascent chain for the various factors, which in turn aid the folding and targeting of the protein client, well documented in the case of trigger factor and SRP. Trigger factor promotes the folding of nascent cytosolic proteins (43), while SRP directs membrane proteins to the translocon (24, 27). But what about SecA and SecB—what's going on?

There are additional anomalies in the literature. For instance, SecA plays a role in SRP-dependent export of soluble protein (44), and the membrane protein RodZ is driven into the membrane by SecA (45), which smells like a cotranslational event. Huber et al. demonstrate conclusively that SecA contacts a variety of nascent proteins *in vivo*, with a clear preference for membrane and secretory proteins (39). This sampling might help prevent aggregation in aid of efficient targeting to the Sec apparatus, but SecA must ultimately be outcompeted from membrane proteins by SRP (Fig. 1A).

An exploration of the interaction with a nascent secretory substrate, the maltose binding protein (MBP), showed that it was independent of trigger factor and SecB. Moreover, the known interaction of SecB (but not trigger factor) with the nascent chain (46) was shown to be dependent on SecA, suggesting that they contact one another at the nascent chain. The association of SecA required a rather long nascent chain (>110 amino acids)—ample space for the independent association of SecA and trigger factor, or SRP for that matter, and for the cooperative association of a SecA-SecB complex.

These new insights compel us to revise the classical overview of bacterial protein secretion and membrane protein insertion to incorporate the action of SecA in cotranslational protein targeting and transport. The authors deal with this neatly by describing a “coupled” and an “uncoupled” cotranslational activity (Fig. 1A and Bii, respectively). In the former, the processive power of protein synthesis, driven by GTP hydrolysis, is coupled to protein translocation from the exit tunnel of the ribosome directly into the translocon and then the membrane during insertion. Uncoupled translocation in this sense refers to the situation where protein synthesis does not drive translocation. In this process, SecA might promote targeting of the nascent chain to the translocon along with SecB. The subsequent association of SecA (and preprotein) to SecYEG would prevent the association of the ribosome, allowing ATP-mediated secretion and, eventually, ribosome dissociation.

The uncoupled cotranslational secretion activity may well preserve some of the features of the posttranslational reaction. For instance, SecA dimer dissociation (47, 48) could promote ATP activation and intercalation of the preprotein (49); interestingly, the structure of the ribosome bound to SecA reveals that both one and two copies can associate (42). Additionally, the formation of a strong interaction with SecB about the unfolded mature regions night help to ensure its efficient transport (Fig. 1Bii). The main advantage, however, would be the increased protection of the substrate from aggregation and the immediate and coordinated targeting of nascent secretory proteins to the translocon.

The availability of alternative posttranslational and uncoupled cotranslational pathways for preprotein secretion (Fig. 1Bi) might be utilized for different kinds of substrates. Small soluble preproteins may well be released from the ribosome before SecA has had a chance to engage the translocon. Larger or more hydrophobic proteins presumably will associate with SecYEG before synthesis is complete. Perhaps even the deployment of rare codons could be used as a mechanism to slow protein synthesis and thereby favor a cotranslational mechanism to help prevent aggregation. Alternatively, there may be shades of gray between true posttranslational translocation and the uncoupled cotranslational process, whereby a cotranslational reaction is initiated and, at some point, protein synthesis is complete and the ribosome drops off. In this case, the transition between co- and posttranslational translocation would depend on the relative rates of protein synthesis and translocation, which will vary respectively and in accordance with codon availability and the protein sequence. We anticipate that some proteins will be translocated much faster than others (50, 51), which will determine whether translocation is mostly post- or cotranslational.

The new results are perhaps a warning that even the classical pathways of yesteryear may need revising. At the same time, they might suggest there are a few more important new aspects of even the Sec machinery that are yet to be revealed.
